# Towards responsible ctDNA-based multi-cancer screening: a preliminary exploration and discussion of ethically relevant aspects

**DOI:** 10.20517/evcna.2022.23

**Published:** 2022-08-16

**Authors:** Wybo Dondorp, Guido de Wert

**Affiliations:** Department of Health, Ethics & Society (HES), School for Oncology and Reproduction (GROW), School for Public Health and Primary Care (CAPHRI), Maastricht University, HA Maastricht 6229, the Netherlands.

**Keywords:** Cancer, population screening, cfDNA, ctDNA, MCED, ethics, public health, screening criteria

## Abstract

While testing for easily accessible biomarkers in the circulation (“liquid biopsy”) has found its way to clinical cancer care, a further expected development is its use as a “universal” early detection test in population screening for cancer. A promising marker for such screening is circulating cell-free fragments of tumor DNA, shed into the circulation during tumor cell turnover. Several blood-based “multicancer early detection (MCED) tests” have recently been developed - but still need validation in large-scale studies involving non-patient populations. In this paper, we proactively explore the ethical aspects of this development. We refer to an often quoted synthesis of the internationally accepted framework of principles for responsible screening as first drawn up for the World Health Organisation (WHO) by Wilson and Junger 50 years ago and further developed and fine-tuned ever since. As our analysis suggests, some specific ethical issues and concerns about potential MCED screening connect to the fact that cancer is not just one disease. As a consequence, not all findings will have the same clinical utility. We discuss this against the background of earlier debates pertaining to broad scope forms of screening in other contexts, specifically newborn and reproductive genetic screening. We highlight the guidance provided by some of the criteria from the screening framework that seems most relevant in this connection: the need for screening objectives to be defined at the outset, the need for mechanisms to minimize potential risks, and the requirement that, for those participating in the screening, the overall benefits outweigh the harm.

## INTRODUCTION

As one of the leading causes of death in developed countries, cancer is a global public health problem. The high mortality burden is a consequence of most cancers being detected at a stage where treatment options have limited effectiveness. Population screening aimed at finding cancer at a pre-symptomatic stage may help change this unfortunate course of events by enabling timely treatment. However, there are many different kinds of cancer and only for some of them have so far made it possible to develop screening strategies for non-patient populations. In the Netherlands, for instance, national screening programs currently only exist for breast, cervical and colorectal cancer, targeting higher-risk age groups (of the relevant sex) in the general population^[1]^. A pilot for lung cancer screening offered to those with a history of heavy smoking (as also recommended in some other countries) has just started^[2]^. For each individual type of cancer, the challenge is to develop a test that is not only easily applicable in pre-symptomatic stages, but also sufficiently accurate and affordable for a population screening program to have a positive benefit to harm and cost-utility ratios. Moreover, with each additional screening for a further type of cancer, logistical burdens and costs will increase and motivation to participate may suffer from screening fatigue, especially in lower-risk populations. 

Against this background, the concept of “universal” (or multi-organ) cancer screening based on easily accessible tumor markers in the circulation may well be revolutionary. This approach would allow simultaneous screening for, in principle, all types of cancer at pre-symptomatic stages using a minimally invasive blood test^[3-5]^. A promising marker for such screening is circulating cell-free fragments of tumor DNA. Also, in healthy individuals, all types of cells release genetic material (cell-free DNA; cfDNA) into the bloodstream during the course of normal cell turnover. This finding has already led to the successful development and implementation in many countries of prenatal screening for fetal chromosomal abnormalities based on the presence of cell-free fetal DNA (cffDNA; actually stemming from the placenta) in the maternal circulation^[6]^. Cell-free tumor DNA (ctDNA) specifically derives from tumor cell turnover. For detection and analysis, state-of-the-art technologies are being used that allow distinguishing the genomic features of the relevant DNA fragments from background signals in the circulation. The idea of using ctDNA, in combination with other biomarkers, as a test for population screening builds on the recent development and clinical use of “liquid biopsy” as a tool in cancer diagnosis, prognosis and treatment, for instance, allowing personalized treatment based on pharmacogenetic profiling, or as a powerful marker in tumor monitoring and follow-up after cancer treatment^[4,7]^. However, using this approach for population screening is more challenging as it requires finding cancer at an early stage in a lower-risk population with sufficient accuracy^[8]^.

Several blood-based “multicancer early detection (MCED) tests” have recently been developed - but still need validation in large-scale studies involving non-patient populations^[5,9]^. The limited evaluations so far suggest a relatively low sensitivity, especially for early-stage cancer^[10,11]^. As a consequence, MCED screening should not be seen as an alternative but as an add-on to current cancer screening programs. Modeling has suggested that this add-on approach might potentially be an efficient cancer screening strategy based on finding additional breast, colorectal, cervical, lung and prostate cancers either missed in current screening programs for those conditions, or in people outside the target population criteria used in those programs or also in those who decline current cancer screening but would agree to MCED testing^[9,12]^. To further evaluate this idea, the GALLERI test is currently piloted for the British NHS. The pilot (2021-2023) comprises a randomized controlled trial for which 140.000 non-patient participants aged 50 to 79 will be recruited^[13]^. The aim is “to establish if screening with the Galleri test reduces the incidence of late-stage cancer when used in an asymptomatic population in combination with existing NHS cancer screening programs”^[14]^.

While the research is still in the test-validation phase, it is also important to pro-actively consider the ethical aspects of this development. In this exploratory paper, we refer to the internationally accepted principles for responsible population screening as a background for further agenda setting. As we will argue, the concept of MCED screening comes with specific ethical challenges that need to be considered. These connect to different ways in which such screening may not only have a potential for benefit but also for harm.

## FRAMEWORK FOR RESPONSIBLE SCREENING

From an ethical perspective, a crucial distinction is between medical testing that is clinically available to patients and those are offered to non-patients in a screening context^[15]^. Whereas clinical testing is performed in response to a complaint or other kind of individual indication on the part of the patient, screening is systematically offered on the initiative of health care professionals or screening authorities to people without a relevant complaint or indication. Leaving aside the special situation of screening in contexts where societal interests justifiably take precedence (for instance, where screening would be necessary as a means to control the spread of a pandemic), the non-indicated nature of screening and the locus of the initiative require a high level of evidence that, on balance, testing is indeed beneficial for at least a significant proportion of those to whom it is offered^[16,17]^.

This would always be the case, which is far from obvious. As famously stated by the former director of the UK National Screening Committee, sir Muir Gray, “[a]ll screening programs do harm; some do good as well, and, of these, some do more good than harm at reasonable cost”^[18]^. Harms of screening may range from psychosocial effects such as induced anxiety or false reassurance to iatrogenic damage as a consequence of overdiagnosis and -treatment. The benefits of screening, mostly understood in terms of health gains, should clearly outweigh these harms. This requires not just that screening outcomes are sufficiently accurate, but that they meaningfully enable altering the clinical outcome in those with positive findings through effective early interventions^[19]^. Moreover, where screening is paid for from public or collective funds, opportunity costs require budgetary justification^[16,17]^.

The acknowledgment of the precariousness of the balance between these different elements is behind the internationally accepted framework of principles for responsible screening, as first drawn up for the World Health Organisation (WHO) by Wilson and Junger in 1968 and further developed and fine-tuned also for specific types of screening (e.g. reproductive and/or genetic screening) by several expert groups and national screening authorities^[17,19]^. An often quoted synthesis of these “emerging screening criteria” was drawn up by Andermann (WHO) and colleagues at the 40th anniversary of the Wilson & Jungner principles in 2008^[20]^ (see Table 1). Highlighting the ethical nature of this internationally endorsed framework, the final and summarizing requirement on their list is that “[t]he overall benefits of screening should outweigh the harm”.

**Table 1:** Screening criteria (Andermann WHO 2008)

**Synthesis of emerging screening criteria proposed over the past 40 years**
• The screening programme should respond to a recognized need.
• **The objectives of screening should be defined at the outset**
• There should be a defined target population
• There should be scientific evidence of screening programme effectiveness
• The programme should integrate education, testing, clinical services and programme management
• **There should be quality assurance, with mechanisms to minimize potential risks of screening**
• The programme should ensure informed choice, confidentiality and respect for autonomy
• The programme should promote equity and access to screening for the entire target population
• Programme evaluation should be planned from the outset
• **The overall benefits of screening should outweigh the harm**
-Reproduced with permission from: Andermann A, Blancquaert I, Beauchamp S, Déry V. Revisiting Wilson and Jungner in the genomic age: a review of screening criteria over the past 40 years. *Bull World Health Organ*. 2008 Apr;86:317-9
-Criteria rendered in bold are specifically discussed in the present text

## RELEVANT ETHICAL ASPECTS OF MCED SCREENING

In a recent review on behalf of the International Society of Liquid Biopsy, Serrano and colleagues explicitly refer to this framework and remark that cancer screening programs based on MCED testing could well fulfill these criteria^[21]^. We agree that there is no apriori reason why this would not be the case, given the large potential health benefits that this approach may yield. However, whether specific screening protocols do indeed meet the criteria for responsible screening and may then (depending on the health system) be either recommended by professional societies and/or incorporated into national screening programs, will have to be determined on the basis of further evidence about relevant factors.

As with all screening programs, test accuracy is an essential quality parameter. A relatively low sensitivity not only affects screening performance; it may also lead to false reassurance. Given that, precisely in the light of lower expected sensitivity, MCED screening would serve as an add-on to, rather than as a replacement for, current single-organ cancer screening programs, there is a potential risk that false reassurance induced by negative blood tests may lead to a lower uptake of current screening. Clearly, this would be an adverse effect of simply adding MCED screening to the current landscape. While such effects can only be determined in actual practice, the remaining importance of current single-organ cancer screening may need to be proactively addressed in the screening information for the relevant target groups. This also suggests that the best way of fitting MCED testing into a wider approach to cancer screening will require more precise attention.

Another ethically relevant issue with test accuracy is the false-positive rate. Even at less than 1% (the rate that some reviewers seem to regard as a benchmark^[9]^), a screening offer to potentially the whole population in a wide age range will lead to many people ending up with a positive MCED test but without radiological or clinical confirmation of cancer. These people will have to be offered further monitoring, for which “pathways should be developed”^[9]^. However, this is more than a logistical issue, as it may induce high levels of anxiety as well as procedure-related harms, especially if the precise focus of the necessary monitoring may not be obvious.

This connects to an important further issue: the screening should not just be able to accurately detect the presence of ctDNA in the circulation, but also to localize the tumor of origin in the correct organ system, ideally using the same blood sample. If not, this might lead to diagnostic delay as well as to unnecessary diagnostic procedures and biopsies, causing psychological distress and possibly also physical harm^[22]^. In order to be able to identify the tumor of origin, MCED tests rely on additional biomarkers beyond ctDNA, such as proteins, or methylation profiles. A recent review of different tests gives rates of accurate tumor of origin identification that at 82%-93% are far from perfect^[9]^. Moreover, this may differ per cancer type. In a study with the CancerSEEK test, predictions were significantly less accurate for lung and liver cancer compared to colorectum and ovary cancer^[23]^.

Moreover, MCED screening may lead to overdiagnosis. This would be the case if screening reveals that part of the cancers are “indolent” rather than a potentially lethal type. Overdiagnosis is problematic as it comes with unnecessary anxiety and may lead to iatrogenic harm due to unnecessary follow-up procedures. The reason why public health authorities in many countries have been reluctant to recommend prostate cancer screening is precisely that the often less aggressive nature of these cancers and the potential for harm make it questionable if such screening would on balance be beneficial. How the proposed MCED tests would relate to the concern about overdiagnosis is as yet unclear. It has been argued that precisely because more aggressive cancers shed more ctDNA into the circulation, findings (including for prostate cancer) will more likely be of a clinically relevant type^[9]^. Even if that mitigates the concern, overdiagnosis may remain a drawback of MCED screening. Clearly, the rejoinder (in a report from the “Information Technology & Innovation Foundation”, a US science and technology policy think tank^[24]^) that “the underdiagnosis of cancers” is a much greater problem than overdiagnosis is not a very helpful response to this concern.

Finally, MCED screening may find cancers for which there are as yet no effective treatment options, such as pancreatic cancer. Early detection of a disease that has no potential of changing the clinical outcome for the patient should be considered a problematic outcome of screening, as in such cases, there are no clear health benefits compensating for the inevitable harms of screening. Especially in a screening context, where tests are proposed on the initiative of health care professionals or screening authorities to people without a relevant complaint or indication, there is every reason to hold on to a more traditional understanding of “clinical utility” as “(referring) to the likelihood that the test will lead to an improved health outcome”^[25]^. This is ignored in rejoinders saying that any health information “no matter how dire the diagnosis” is always useful, or that the patient is bound to find out anyway and that “it matters little whether that that knowledge is delivered earlier” as a consequence of MCED screening, or that the screening might contribute to developing those very therapies for future patients^[24]^.

## DISCUSSION

As Putcha *et al*. have commented, “[c]ancer sounds like one disease, but is actually many: there are more than 100 different cancers, each with multiple subtypes reflecting different underlying molecular pathophysiologies”^[22]^. This means that what is presented as “universal” cancer screening is not so much “screening for cancer, period”, but rather screening for a broad range of disorders with different clinical profiles using one simple test, based on the fact that all cancers happen to shed ctDNA in the circulation. Clearly, this renders the evaluation of MCED screening programs more complex than where concerning screening for one specific form of cancer, as it need not be the case that for each and every type of cancer that the test might detect, the benefits clearly outweigh any possible harms. While the overall benefit-to-harm ratio of MCED screening may well be positive (depending on further evidence from validation studies and pilots), some possible outcomes may represent a moral cost that one would rather avoid.

This is not to say that, in terms of debates about screening ethics, we are entering uncharted territory. Whereas the MCED approach is hailed as a “revolution” in cancer screening, the concept of multi-disorder screening as such is not entirely new, nor is the debate about its ethical implications. For instance, in newborn screening (NBS) for metabolic disorders, the use of tandem mass spectrometry (MS/MS) was introduced in the 1990s, enabling the simultaneous accurate and cost-effective measurement of large numbers of metabolites in dried blood spots on “heelprick” screening cards. As commented in a review, this “has deeply changed the older NBS approach of “one test for one disorder” to “one test for many disorders”, i.e., a multiplex test”^[26]^. Current newborn screening programs worldwide use this technology, typically screening for several tens of inherited metabolic diseases^[27]^. More recently, we have seen the development of broad scope genomic tests that have significantly changed the field of prenatal screening (based on cfDNA in maternal blood)^[28]^; they are piloted for preconception carrier screening programs^[29]^ and are expected to further impact the practice of NBS in the years to come^[30]^.

In each of these contexts, the perspective in the debate about the scope of screening has changed from: “what to test for?” to: “what conditions, if any, to actively exclude from the results of broad scope testing”^[16,31]^? For instance, in newborn screening, many commentators would want to hold on to the idea that neonates should not be screened for diseases for which there is no meaningful treatment or prevention, or for diseases that would only manifest later in life and the course of which cannot be altered by preventative measures taken at a young age^[32,33]^. This would require using “filters” so as to avoid any unsolicited findings coming available as a result of a comprehensive genetic screening test. However, others have questioned this approach, stressing, e.g., that parents have a right to health information about their children, or that families may benefit from information relevant for future reproductive choices, or also that finding diseases for which there are presently no therapeutic options, would still be useful as it would contribute to research that might lead to developing treatments, in the interest of future children with the same disorder, as well as of society at large^[34,35]^. Similarly, while professional societies do not recommend analyzing cfDNA-findings from prenatal screening also for sex chromosome aneuploidies (SCA), pointing to low predictive value, generally mild phenotypes, and uncertain clinical utility^[36]^, commercial screening offers often do include testing for these abnormalities, with advocates reasoning that it is for the woman to decide if such findings would be relevant for reproductive decision-making. A recent Australian study reports on how this has led to a steep increase in prenatal diagnosis for SCA since the introduction of cfDNA-based prenatal screening^[37]^. 

In our view, it is difficult not to recognize the undercurrent of technology-driven justifications for ever wider screening in these debates, with the fact that we *can* test for something being taken as a sufficient reason as to why we should^[38,39]^*. *As the framing of “universal” cancer screening may well fit in with this “find everything we can” perspective, it is crucial to timely address the arguments relevant to what the scope of the envisaged screening should be. Referring to the above framework, we think the following three criteria from the Andermann list are especially important^[20]^.

Firstly, “[t]he objectives of screening should be defined at the outset”. What is it that we may want to achieve with MCED screening? Is it accurately detecting cancer signals in the circulation, is it enabling the diagnosis of early-stage cancers that are now often missed, or is it early diagnosis leading to meaningfully changing the clinical outcome in people with different forms of cancer? It seems quite clear that in the context of a public health service, only the latter answer will do. But then, some of the potential outcomes listed in the previous section (such as problems finding the tumor of origin, overdiagnosis, or finding cancer for which no meaningful treatment options are available) should be regarded as at odds with the screening objective and, in so far, problematic. It is important for the screening objective to be “defined at the outset”, in order to enable proper evaluation of ongoing screening programs.

Secondly, “[t]here should be (…) mechanisms to minimize potential risks of screening”. This is especially important in view of the fact that broad scope screening may inevitably yield outcomes at odds with the screening objective. One mechanism of minimizing any resulting harm for MCED screening participants might be the use of filters to avoid such “unsolicited findings”. This may take the form of using specific cut-offs in ctDNA-analysis to limit overdiagnosis, but perhaps also of using tumor of origin information with an eye to avoid finding forms of cancer without meaningful treatment options. The need for specific measures of this type would, of course, need justification in the light of the screening objective. Where such measures are not feasible, the potential for harmful “unsolicited findings” should be included in the weighing of the overall benefit-to-harm ratio of the screening program. Where screening programs with a risk of such outcomes are offered, proper information and counseling are needed, also in order to protect participants from ending up in a trap that they would rather have avoided.

That brings us, thirdly, to the summarizing ethical requirement that “[t]he overall benefits of screening should outweigh the harm”. In the light of our above discussion (and apart from pandemic-type emergency situations), it would seem necessary to specify this as referring to the benefits and harms “for those participating in the screening”^[17]^. This is not to say that screening might not also be beneficial for family members, future patients, or society at large. Indeed, most screening programs are meant to achieve health gains at a population level^[16]^. However, it is to say that third party benefits should not be presented as outweighing the harms of specific forms of screening that would not clearly also benefit the participants themselves. This is especially relevant in view of the potentially high research value of the data generated through powerful new broad-scope screening technologies, including MCED^[24]^. Without the specification of who is on balance to benefit, there is a risk in these developments of blurring the boundary between screening and research, turning screening participants into research subjects without proper safeguards^[31]^.

## CONCLUSION

The prospect of ctDNA-based early detection of cancer may well change the field of cancer screening as we know it, with potentially large public health and individual benefits. While validation studies of MCED tests are only starting, it is important to pro-actively also consider the ethically relevant aspects of this development. In this connection, it is important to be aware that cancer is not just one disease and that not all findings will therefore have the same clinical utility. As forms of multi-disorder screening have been introduced in other contexts, the ethical analysis may connect to relevant earlier debates about how to deal with specific challenges of optimizing the benefit-to-risk ratio of such forms of screening. As we have argued, the traditional framework for responsible screening still seems to provide useful guidance in this respect.

## DECLARATIONS

### Copyright

© The Author(s) 2022.
